# A Microfluidic Platform Integrating Dielectrophoretic Concentration and Impedimetric Sensing for Rapid *Salmonella choleraesuis* Detection

**DOI:** 10.1021/acssensors.5c00779

**Published:** 2025-09-18

**Authors:** Avinash V Police Patil, Yu-Sheng Chuang, Che-Wei Lin, Chiou-Ying Yang, Tomoyuki Yasukawa, Ching-Chou Wu

**Affiliations:** † Department of Bio-Industrial Mechatronics Engineering, 34916 National Chung Hsing University, Taichung City 402, Taiwan; ‡ Institute of Molecular Biology, National Chung Hsing University, Taichung City 402, Taiwan; § Graduate School of Science, University of Hyogo, Ako 678-1297, Japan; ∥ Innovation and Development Center of Sustainable Agriculture, National Chung Hsing University, Taichung City 402, Taiwan

**Keywords:** immunomagnetic beads, electrochemical impedance spectroscopy, dielectrophoresis, microfluidics, Salmonella

## Abstract

Fast and sensitive detection of foodborne bacteria, such as *Salmonella*, is essential for food safety and public health control. This study constructed a microfluidic device integrating a top-bottom opposite electrode pair for performing positive dielectrophoresis (pDEP) and electrochemical impedance spectroscopy (EIS). The immunomagnetic beads (IMBs) modified with anti-*Salmonella* antibodies were spiked in *Salmonella enterica* serovar Choleraesuis (*SC*)-containing samples for 30 min to form IMBs@*SC* complexes, and they were separated by a magnet, which can prevent the sample matrix from contaminating the EIS detectors. After applying the 50 kHz/7.5 V_pp_ pDEP for 3 min to collect the IMBs@*SC* complexes in the microholes of the working electrodes, EIS was used to determine the *SC* concentration. The linear range and the calculated detection limit obtained from *SC*-spiked undiluted milk samples are 10^1^ to 10^4^ and 2 cfu/mL. The total procedures from the IMBs immunoreaction to the sample-to-result on the device only take 50 min. The microfluidic device offers a promising platform for the rapid and ultrasensitive detection of pathogenic bacteria.

## Graphical abstract

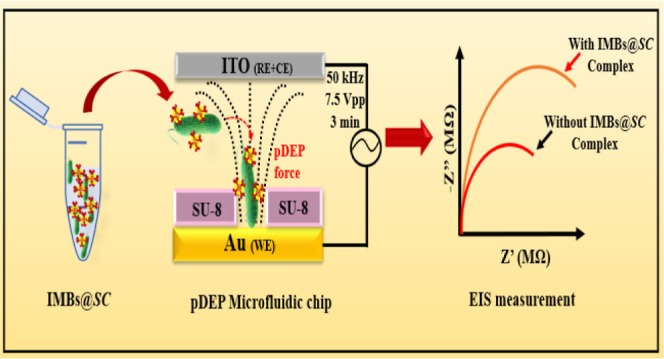

Foodborne pathogens cause major public health risks and significant economic losses globally. The World Health Organization identifies *Salmonella* *spp*. as one of the most common foodborne pathogens for foodborne illnesses worldwide, and it cannot be detected in ready-to-eat foods.^1–4^ *Salmonella* can contaminate food products, such as eggs, undercooked meats, milk, and juices and cause symptoms like vomiting, diarrhea, and severe cases, leading to bacteremia or bacteriuria.^1–4^ These conditions are life-threatening if *Salmonella* is not quickly identified and effectively treated. Therefore, developing ultrasensitive and rapid methods for detecting *Salmonella* without preculturing procedures is crucial for food safety and clinical diagnostics.

Several methods have been employed to detect *Salmonella* in food matrices, including the conventional culture method (ISO 6579-1:2017),^5,6^ nucleic acid amplification tests (NAATs), such as polymerase chain reaction (PCR),^7–10^ and loop-mediated isothermal amplification, and immunoassays,^11–13^ such as enzyme-linked immunosorbent assay (ELISA),^14–16^ lateral flow immunoassay (LFIA),^17,18^ and biosensor techniques.^19–23^ Conventional agar-plate culture methods take several days for bacterial growth, which is time-consuming and labor-intensive. Generally, NAATs need elaborate extraction procedures, expensive reagents, and suitable temperature-controlled reactors for nucleic acid amplification. Commercial ELISA and LFIA typically take at least 6 h for bacterial proliferation due to the sensitivity limitation before signal detection.^24–27^ It is meaningful for developing more sensitive and quick enrichment techniques for urgent detection without long bacterial proliferation.

In contrast, label-free electrochemical affinity-based biosensors, such as immunosensors^5,28,29^ and aptasensors,^30–34^ have been constructed to offer rapid, quantitative, and sensitive bacterial detection, making them more effective for timely and accurate response to *Salmonella* outbreaks. Mutreja et al. constructed graphene–graphene oxide-modified screen-printed carbon electrodes for antibody immobilization.^35^ The electron transfer resister value (*R*_et_) computed from electrochemical impedance spectroscopy (EIS) was used to quantify *Salmonella typhimurium* (*S. Typhimurium*) immunoreaction with a calculated limit of detection (LOD) of 10 cfu/mL in spiked juice samples. Li et al. synthesized Fe_3_O_4_-ionic liquid nanocomposites as a surface modifier of gold electrodes to enhance electron transport and the antifouling ability of the immunosensors against milk contamination. Differential pulse voltammetry (DPV) was utilized to quantify *S. Typhimurium* concentration with a 10^2^ cfu/mL LOD within 40 min without the preculture.^36^ Gong et al. modified a glassy carbon electrode with polyxanthurenic acid, polydopamine, and chondroitin sulfate for aptamer immobilization with antifouling improvement for milk and juice samples. The DPV-based aptasensors can detect *S. Typhimurium* in the linear range of 10^1^–10^7^ cfu/mL and a calculated LOD of 3 cfu/mL in 60 min*.* ^37^ These results elucidate that electrochemical affinity-based biosensors have promising sensitivity for *Salmonella* detection. However, these biosensors require adequate immobilization of antibodies or aptamers and antifouling modification to reduce biosample interference. Moreover, they also require an enzyme- or nanomaterial-labeled detection antibody (DAb) to amplify target signals. The complicated preparation of these biosensors and washing procedures after immunoreaction increase the cost and operation time.^2,23^

Furthermore, adopting magnetic beads (MBs) to collect targets is another effective strategy to reduce biosample contamination in electrochemical biosensors. Their large surface-to-volume ratio, high reaction kinetics, and the feasibility manipulated by external magnetic and electric fields render them ideal antibody-modified carriers for bacterial capture from biological samples^38–41^ or electrochemical signal reporters.^38,42,43^ Xu et al. synthesized capture antibody (CAb)-modified immunomagnetic beads (IMBs) to separate *S. Typhimurium* and then immunoreacted with the glucose oxidase (GOx)-conjugated DAb. The collected IMBs/*Salmonella*/GOx-DAb complexes reacted with glucose to produce gluconic acid and increase the solution conductivity. The LOD of *S. Typhimurium* was obtained at about 10^3^ cfu/mL in 2 h. The manual procedures of preparing the IMBs/bacteria/GOx-DAb complexes and enzymatic catalysis are complicated and tedious.^41^ In contrast, Xue et al. combined a magnetic stirring mixer, a magnetic separation channel, and an impedimetric detector in a microfluidic device to accelerate the immunoreaction and collection of the IMBs/bacteria complexes.^40^ IMBs and the GOx-DAb-modified polystyrene beads (PSB) immunoreacted with *S. Typhimurium* to form IMBs/*S. Typhimurium*/PSB complexes. After collecting the complexes in the magnetic separation channel, a glucose solution was injected to produce gluconic acid. The impedimetric detector quantified the solution conductivity with the increasing *S. Typhimurium* concentration and obtained a 53 cfu/mL LOD in 1.5 h.

Dielectrophoresis (DEP) collection is another effective technique for enriching bacteria and cells in microfluidic devices.^44^ Through nonhomogeneous electric fields, the positive DEP (pDEP) force can attract the higher polarization particles than the medium toward higher electric field regions.^45^ Furthermore, Cheng et al. constructed a microfluidic chip with top-bottom opposite electrodes to produce a negative DEP (nDEP) trap for bacteria collection.^46^ However, this study did not combine any detectors on the same substrate. Jung et al. integrated three components in a microfluidic device for the magnetophoretic concentration and separation of IMBs/*Staphylococcus aureus* complexes via a permanent magnet from food samples, the complex separation from the free IMBs via a pDEP electrode array, and the impedimetric detection of the complexes through two parallel electrodes, respectively.^39^ Eventually, the LOD was 36 cfu/mL after 60 min operation. This device adopts a trap-detection-and-release strategy and the impedimetric detectors without CAb immobilization, which is feasible for consecutive detection after regeneration. However, most DEP-collected complexes were gathered on the edge of the planar parallel electrode (high electric field), which cannot effectively block the entire electrode surface, causing a small impedimetric change. The study inspires us to improve the design of the microfluidic chip combining DEP and impedimetric electrodes to reach more sensitive and faster bacterial detection.

In this study, anti-*Salmonella* CAb (7F1A)-modified IMBs are prepared for *Salmonella enterica* subsp. *enterica* serovar Choleraesuis (*SC*) immunoreaction in standard buffer and undiluted milk samples. Then, the IMBs@*SC* complexes are suspended in pure water after magnetic collection. The IMBs@*SC* solution is conducted in the pDEP-based microfluidic device integrated with the top-bottom opposite electrodes. Several tens of 10 μm diameter microholes are set at the working electrode’s (WE) surface to generate pDEP forces for the IMBs@*SC* capture. Then, EIS is subsequently performed to quantify the *SC* concentration. The adequate frequencies and application times of pDEP are discussed in detail to enhance the IMBs@*SC* enrichment.

## Materials and Methods

### Reagents

Carboxylic acid-modified Fe_3_O_4_ MBs with a hydrodynamic diameter of 100 nm were purchased from Chemi-cell (fluidMAG-CT). Sulfuric acid, hydrochloric acid, nitric acid, ethyl-3-(3-dimethylamino-propyl) carbodiimide HCl (EDC), *N*-hydroxysuccinimide (NHS), 2-[*N*-Morpholino] ethanesulfonic acid (MES), sodium phosphate dibasic (Na_2_HPO_4_), sodium phosphate monobasic dehydrate (NaH_2_PO_4_), 37% formaldehyde, and bovine serum albumin (BSA) were purchased from Sigma-Aldrich. Potassium hexacyanoferrate (III) (K_3_[Fe (CN)_6_], FIC) and potassium hexacyanoferrate (II) trihydrate (K_4_[Fe (CN)_6_]·3H_2_O, FOC) were purchased from Showa. All chemicals were used as reagent grade without further purification. The co-author, Y. Yang, provided *SC* and the *anti-SC* CAb (7F1A). The specificity test and immunoreactivity of 7F1A CAb are described in the Supporting Information S2.1. The results of Figures S1–S3 show that the 7F1A CAb exhibits a broad immunoreactivity for different *Salmonella enterica* subsp. *enterica* serovars and a reasonable specificity without immunoreactivity for *Escherichia coli* *.* The preparation of inactivated *SC* via formaldehyde fixation for biosafety control is mentioned in the Supporting Information S2.2. The results of Figure S4 indicate that the 7F1A CAb effectively recognizes *SC*, regardless of formaldehyde fixation, making it a suitable candidate for immunodetection applications.

Phosphate buffer solution (PBS, pH 7.0) was prepared with 10 mM NaH_2_PO_4_ and 10 mM Na_2_HPO_4_ and used as a background solution for preparing antibodies, bacteria, and electrochemical measurements. The 5 mM equimolar [Fe­(CN)_6_]^3–/4–^ mediators were prepared in 10 mM PBS (pH 7.0), named PBS­(FIC/FOC), for electrochemical detection. All solutions were prepared with water purified through a Milli-Q system (18.2 MΩcm).

### Preparation of IMBs and IMBs@*SC* Complex

IMBs were prepared according to our previous study.^38,47^ 5 μg MBs were suspended in a 95 μL aliquot of 30 mM EDC/NHS-containing MES solution (100 mM, pH 4.6) by shaking at 60 rpm for 30 min at room temperature (RT) to activate the carboxyl group of MBs. A magnet collected the activated MBs for 5 min, washed twice with 150 mM NaCl-containing PBS, and then redispersed in 90 μL of PBS. Subsequently, a 10 μL aliquot of 1 mg/mL Ab was added to form IMBs with a shaker at 60 rpm for 30 min at RT. After collecting the IMBs with a magnet, the IMBs were suspended in 95 μL of PBS to keep the IMBs concentration at 2 × 10^8^ particles/μL and stored at 4 °C for use. A 100 μL aliquot of concentration-varied *SC* solutions ranging from 10^1^ to 10^5^ cfu/mL was added with the 2.5 μL of IMBs solutions with 60 rpm shaking for 30 min at room temperature. A magnet isolated the IMBs@*SC* complexes for 5 min and then washed twice with PBS containing 150 mM NaCl. Finally, the IMBs@*SC* complexes were suspended in 100 μL of DI water for subsequent DEP capture, as depicted in Figure S5 (Supporting Information S2.3).

### Design and Fabrication of the Microfluidic Chip

The microfluidic device, as illustrated in Figure 1A, consists of two poly­(methyl methacrylate) (PMMA) slabs, two recess-containing chip holders, a hollow silicone adhesive tape, an indium tin oxide (ITO) chip, and a working electrode (WE) chip. The chip holders were made by a photocuring 3D printer (Form3B, Teama, Taiwan). The top chip holder has a recess for fixing the ITO chip and two holes for the inlet and outlet of the silicone tape channel. The bottom chip holder contains a recess for the fixation of the WE chip to form the bottom of the silicone-type channel. The seams between the recesses of the chip holders and the chips were sealed with waterproof glue to prevent the solution from leaking. A CO_2_ laser cutter (Beamo, Flux Inc., Taiwan) ablates the ITO layer of the ITO chip into two parts to form a pseudoreference electrode (RE) and a counter electrode (CE). Microfabrication techniques patterned three Au electrodes on the WE chips sputtered with the 200 nm Au/20 nm Ti layers. Each Au electrode surface was covered with a SU-8 3010 negative photoresist layer (MicroChem, Newton, MA) of about 10 μm thickness to produce a microhole array, as shown in Figure 1B. Each WE had the microhole arrays of 10 columns. Each column contains 15 microholes. The interval between the two microhole columns and each column’s two microholes was 150 and 160 μm, respectively. The diameter of each microhole was about 10 μm. The 60 μm thick silicone tape was cut with the CO_2_ laser cutter to form a 40 mm long and 2 mm wide channel. Subsequently, the two PMMA slabs were used to clamp the assembly of the ITO&Au chip-fixed holders and the silicone tape to form the fluidic channel, as shown in Figure 1C. Moreover, the ITO-CE region was face-to-face parallel to the WE chip to form the top-bottom opposite electrodes for DEP manipulation and EIS measurement.

**Figure 1:**
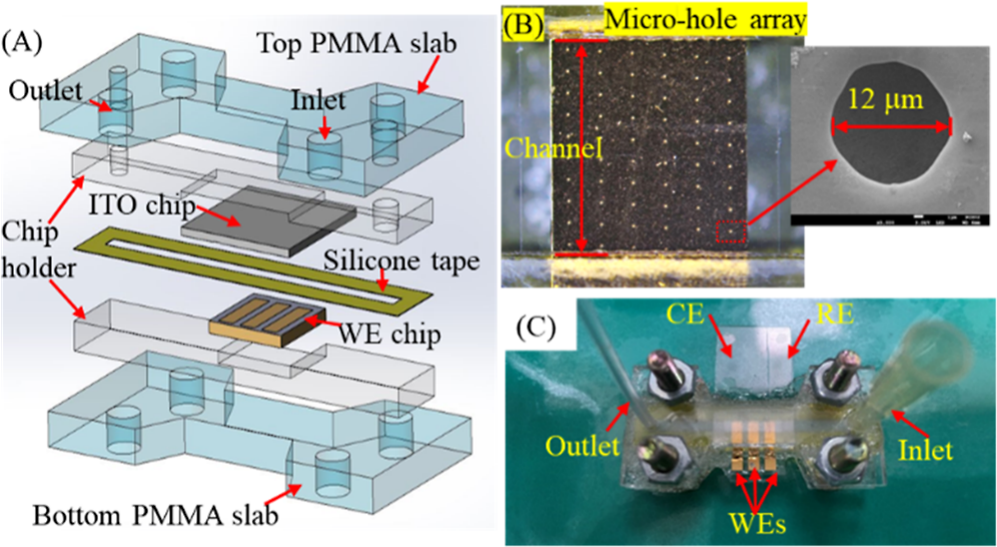
Schematic representation of the fluidic device integrating the top-bottom opposite electrodes. (A) Exploded view of the fluidic device. (B) The optical image of the single Au WE and the SEM image of a microhole on the WE surface. (C) Actual image of the device after assembling different components.

### Operation of the Fluidic Device with pDEP Control

The outlet of the fluidic device was connected to a tube and a syringe pump to control the flow rate and volume of the sample solution. Initially, a 100 μL aliquot of distilled water was dripped into the inlet container and then conducted into the channel at a 25 μL/min flow rate. Subsequently, an AC voltage of 7.5 V_pp_ at 50 kHz produced with a function generator (33220A, Keysight Technologies Inc., California, U.S.A.) was applied on the CE and the WEs for 5 min to activate the Au surface of WEs. Then, the distilled water was replaced by 100 μL of PBS­(FIC/FOC) solution for EIS measurement to obtain the background *R*_et_ value, named *R*_et0_. The adequate activation time of 7.5 V_pp_ electrochemical cleaning is explored in Figure S6 and Table S2 (Supporting Information S2.4).

A 100 μL aliquot of concentration-varying IMBs@*SC* complexes flowed into the channel with a 25 μL/min flow rate. Simultaneously, a 7.5 V_pp_ AC voltage of 50 kHz was provided with the function generator via a power amplifier (HA-405, Pintek Electronics Co., Ltd., Taiwan) to induce pDEP behavior. The DEP force of particles suspended in a medium under the action of nonuniform electric fields can be described as eq 1 ^45^

1 $$F_{DEP}=2\pi \varepsilon_{m}r^{3}Re\left(f_{CM}\right)\nabla E^{2}$$

where ε_m_ represents the relative permittivity of the medium, *r* is the radius of the IMBs@*SC* particle, Re­(*f*_CM_) is the real part of the Clausius–Mossotti (CM) factor, and *E* is the amplitude of the electric field. Re­(*f*_CM_) determines the *F*_DEP_ vector of the particles. When Re­(*f*_CM_) is positive, the particles are driven to a high electric field. Moreover, the *f*_CM_ represents the particle’s polarizability, as mentioned in eq 2

2 $$f_{CM}=\frac{\varepsilon_{p}^{*}-\varepsilon_{m}^{*}}{\varepsilon_{p}^{*}+2\varepsilon_{m}^{*}}$$

where ε_p_* and ε_m_* represent the complex permittivity of particle and medium, respectively, and ε* = ε – *i*(σ/ω), where σ is the conductivity and ω­(=2π*f*) is the angular frequency. Referring to the previous paper,^48^ the IMBs@*SC* complexes have higher conductivity than DI water (0.055 μS/cm) due to the highly conductive MBs (2.99 mS/cm). In this study, the IMBs@*SC* complexes were suspended in DI water to confirm pDEP generation to drive the IMBs@*SC* to the high electric field region of microholes.

After capturing the IMBs@*SC* complexes, 100 μL of PBS­(FIC/FOC) solution was conducted in the channel for the EIS measurement to obtain the *R*_et_ signal of IMBs@*SC* complexes. Finally, the device was inverted, and a 100 μL aliquot of DI water flowed into the channel with a 100 μL/min flow rate for chip cleaning. During flow, the bottom of the device was gently tapped by a fingertip to facilitate the movement of IMBs@*SC* from the microholes for sensor regeneration. The whole steps of device operation for SC detection and EIS detector regeneration are shown in Scheme 1.

**Scheme 1:**
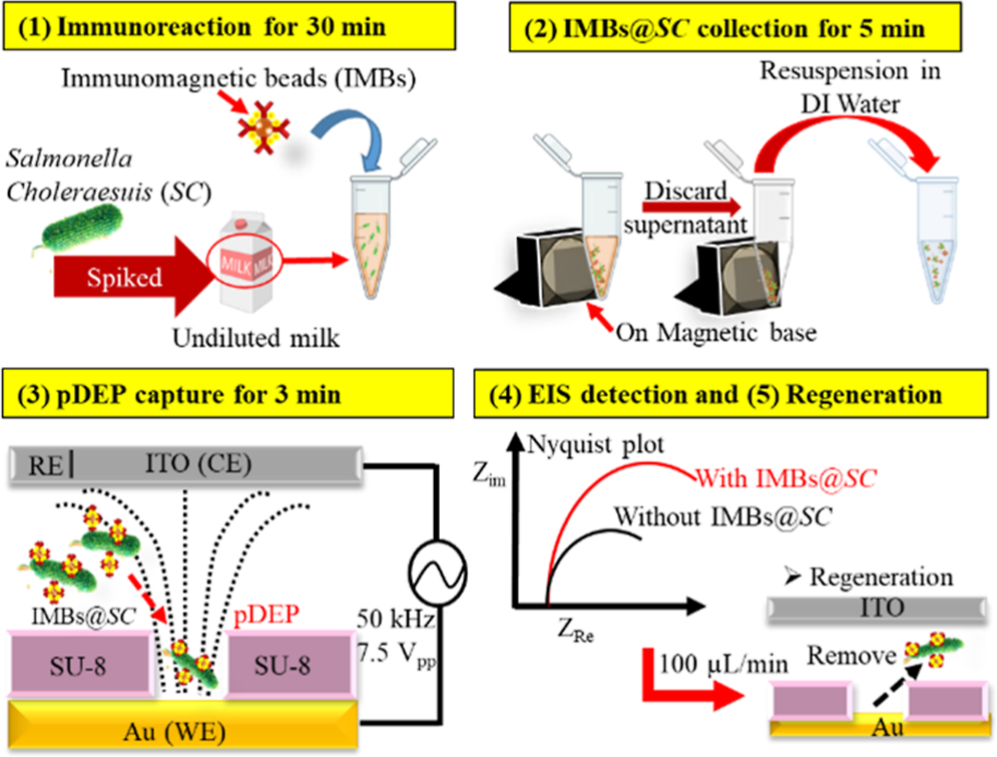
Schematic of an Immunomagnetic Bead-Based EIS Sensor with DEP and Microfluidic Control for Detecting *SC* in Milk with the 1–5 Sequential Steps

### Electrochemical Measurements

All electrochemical measurements were conducted using an IM-6 impedance analyzer (Zahner Electrik GmbH, Germany) in a three-electrode configuration with the ITO RE, the ITO CE, and the Au WE. The 5 mM equimolar [Fe­(CN)_6_]^3–/4–^ mediators were employed to probe the electrochemical properties of the WE surface. EIS measurements were performed with a frequency range of 1 Hz to 100 kHz at 0 V potential (versus the ITO RE) with a 5 mV amplitude sine wave.^48^ The IM-6/THALES software package was utilized to analyze the impedance spectra and simulate different equivalent circuits. Referring to our previous papers,^38,49^ using the modified Randles equivalent circuit, called the 1*R*//*C* circuit, consisting of a solution resistor (*R*_s_) in series with a parallel combination of a *R*_et_ and a constant phase element (CPE), explains the electrochemical properties of the electrode/electrolyte interface. The combination of *R*_et_ and CPE represents the electron transfer kinetic-controlled behavior, which is influenced by the interactions between the electrode and the IMBs@*SC*. The CPE accounts for the nonideal capacitive phenomenon of the inhomogeneous electrode surface.

### Data Processing and Statistical Analysis

All experiments were performed in triplicate, including measurements of blank samples. Data are reported as mean values with standard deviations (mean ± SD). Calibration curves were constructed by plotting the measured signal against the logarithm of the *SC* bacterial concentration. The LOD was calculated according to the formula LOD = 3 × (SD/*m*), where SD represents the standard deviation of the blank measurements and *m* denotes the slope of the calibration curve. Statistical analysis and data fitting were performed by using Microsoft Excel.

## Results and Discussion

### Optimization of the IMBs-to-*SC* Ratio

The adequate ratio of IMBs to *SC* numbers is beneficial for effectively collecting the IMBs@*SC* complexes from the samples after using a magnet and reducing the IMBs usage cost. Figure 2 shows the SEM images of glass substrates coated by a 20 μL aliquot of the supernatant (A–C) and the collection (A′–C′), respectively, after using a magnet to collect the IMBs@*SC* complexes, which are immunoreacted with the ratios (100:1, 500:1, and 1000:1) of IMBs to *SC* numbers (10^7^ cfu/mL). The results show that the IMBs-to-*SC* ratios of 500:1 and 1000:1 can obtain more IMBs@*SC* complexes in the collection than the 100:1 ratio. The 500:1 ratio was used for the pDEP enrichment in the microfluidic device to save IMBs usage.

**Figure 2:**
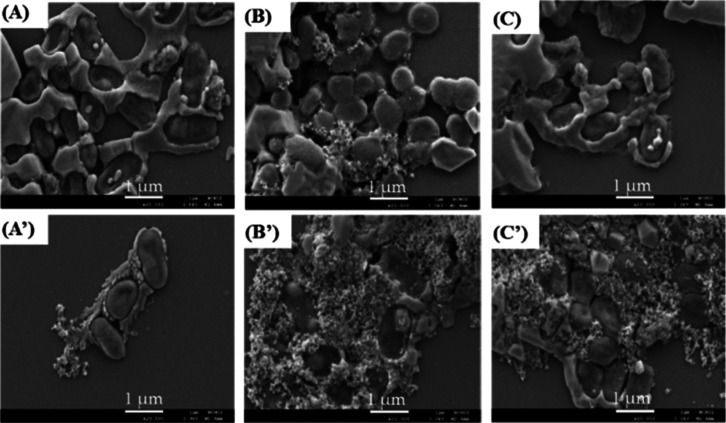
SEM images of glass substrates coated by the supernatant (A–C) and the collection (A′–C′) solutions after using a magnet to collect the IMBs@*SC* complexes for 5 min. Three ratios, 100:1 (A,A′), 500:1 (B,B′), and 1000:1 (C,C′), of IMBs to *SC* are immunoreacted.

### Effect of pDEP Frequencies

Two forces, *F*_DEP_ and the viscous drag force (*F*_η_), simultaneously determine the capturing efficiency of IMBs@*SC* complexes. The IMBs@*SC* particles can be captured into the microholes when the component of the *F*_DEP_ perpendicular to the microhole direction exceeds the *F*_η_. *F*_DEP_ is proportional to the IMBs@*SC* size, Re­(*f*_CM_), and ∇*E* ^2^ values. Figure S7 (Supporting Information Figure S3.1) shows the numerical simulation images of electric fields. The result indicates that a nonuniform electric field can be distributed in a 60 μm high microchannel, and the maximal electric field gradient occurs at the inner edge of microholes. The result implies that the IMBs@*SC*, with a larger conductivity than DI water, can be driven to microholes due to the pDEP force.

Furthermore, the *f*_CM_ of the pDEP force is determined by electric frequencies and the σ and ε of the particle and medium (eq 2). Theoretically, when the frequency approaches the relaxation frequency, the Re­(*f*_CM_) decreases with the increasing frequency, causing a smaller pDEP force.^45^ In this study, we kept a constant flow rate, equivalent to *F*_η_, and explored the effect of the frequency-varied 7.5 V_pp_ pDEP on IMBs@*SC* capture. Figure 3 shows the effect of the frequency-varying 7.5 V_pp_ pDEP on the IMBs@*SC* capture. A 100 μL aliquot of IMBs@*SC* (10^5^ cfu/mL) solution was conducted at a 25 μL/min flow rate, and the frequency-varied 7.5 V_pp_ was applied for 1 min. Subsequently, 100 μL of PBS­(FIC/FOC) replaced the IMBs@*SC* solution with the 25 μL/min flow rate for in situ EIS measurement. Figure 3a shows the Nyquist plots obtained from frequency-varied DEP. The Nyquist plot of 50 kHz pDEP has the maximal semicircular radius, implying a more significant *R*_et_ value than the other frequency pDEP.^49^ After simulating the Nyquist plots by the 1*R*//*C* equivalent circuit,^49^ the *R*_et_ increment ratio (=Δ*R*_et_/*R*_et0_, where Δ*R*_et_ = *R*_et–pDEP_ – *R*_et0_) was used to indicate the number of the captured IMBs@*SC* complexes, as shown in Figure 3b. The result shows that when the pDEP frequency exceeds 50 kHz, the Δ*R*_et_/*R*_et0_ declines, suggesting a smaller pDEP force and *f*_CM_. Therefore, a 50 kHz 7.5 V_pp_ potential was used for subsequent experiments.

**Figure 3:**
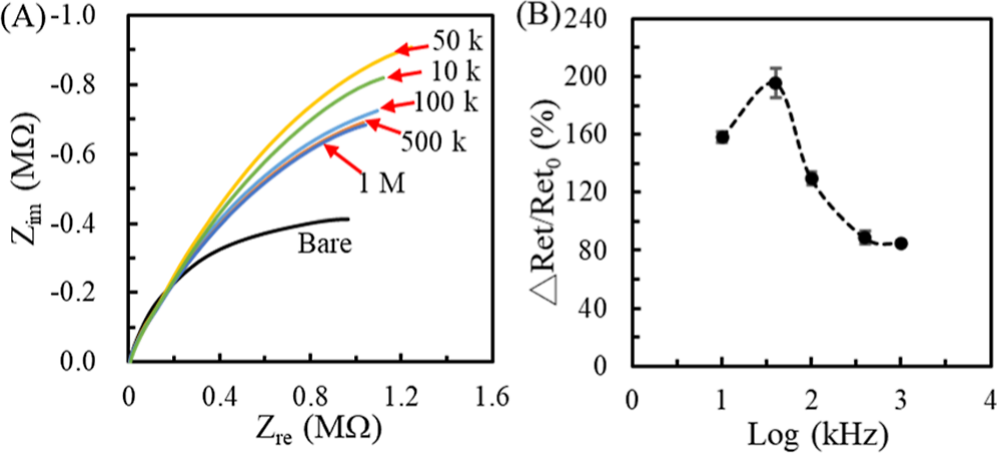
Changes on Nyquist plots (A) and Δ*R*_et_/*R*_et0_ (B) after conducting the IMBs@*SC* (10^5^ cfu/mL) solution with a 25 μL/min flow rate and the frequency-varied (10, 50, 100, 500, and 1000 kHz) 7.5 V_pp_ for 1 min.

### Sensing Properties with 1 min DEP Collection

This study proposes a rapid, regenerable microfluidic sensing platform for specific bacterial detection. Utilizing IMBs for specifically immunoreacting *SC* can prevent the biomatrix from contaminating the sensing electrode. Moreover, the WEs without the antibody’s immobilization can benefit from massive production via microfabrication techniques and readily regenerate using a fluidic shear force. Figure 4A shows the EIS signals after conducting 100 μL concentration-varied IMBs@*SC* samples (10^2^–10^5^ cfu/mL) in the microfluidic device with the 1 min DEP collection. The semicircle radius of Nyquist plots increased with the *SC* concentration, indicating that the number of IMBs@*SC* complexes captured on the microhole WE increased. Figure 4B shows the calibration curve in the range of 10^2^–10^5^ cfu/mL with the regressive equation of Δ*R*_et_/*R*_et0_ (%) = 45.182 log­[*SC*]­(cfu/mL) – 65.808. The calculated LOD was 44 cfu/mL (S/*N* > 3).

**Figure 4:**
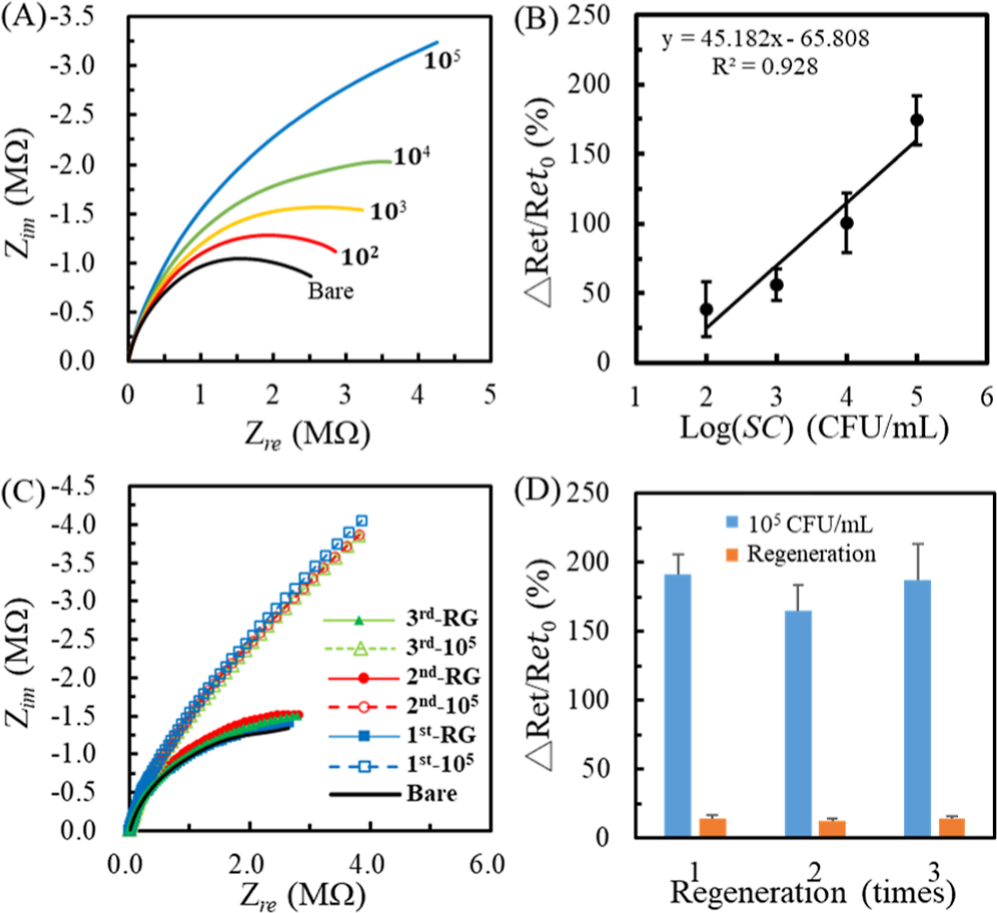
(A) The Nyquist plots of concentration-varied IMBs@*SC* solutions with the 1 min DEP collection at a 25 μL/min flow rate. (B) The calibration curve corresponds to (A). (C) The Nyquist plots before and after repeating the DEP collection of IMBs@*SC* (10^5^ cfu/mL) and regenerating the chip thrice. (d) The Δ*R*_et_/*R*_et0_ values correspond to (c). Each statistic data is calculated from three individual WEs.

Figure 4C,D shows the effect of regeneration processes on the Δ*R*_et_/*R*_et0_ after conducting the IMBs@*SC* (10^5^ cfu/mL) solution with 1 min pDEP collection. The Nyquist plots exhibited higher reproducible results after repeating the pDEP collection and flow regeneration three times. The average Δ*R*_et_/*R*_et0_ value was 181 ± 14% after the DEP collection. The relative standard deviation (RSD) was 7.7%, implying that the sensing device has good reproducibility. Moreover, the Δ*R*_et_/*R*_et0_ was 14 ± 1% after the flow regeneration, significantly smaller than the detection signal. This study proves the distinct advantage of reusing the WEs and microfluidic devices due to the little residue from former detection.

### Effect of DEP Time on the Sensing Properties

In principle, the longer DEP application time can capture more IMBs@*SC* numbers in the microholes. Figure 5A,B, respectively, shows the Nyquist plots and their Δ*R*_et_/*R*_et0_ after conducting 100 μL of IMBs@*SC* (10^2^ cfu/mL) with 1, 2, or 3 min pDEP collection under the 25 μL/min flow. The semicircle radius of the Nyquist plots increases with the DEP time, suggesting an increasing *R*_et_. The Δ*R*_et_/*R*_et0_ increased from 41.9 ± 0.5% to 105.3 ± 4.4%, indicating that the longer DEP applications can collect more IMBs@*SC* complexes.

**Figure 5:**
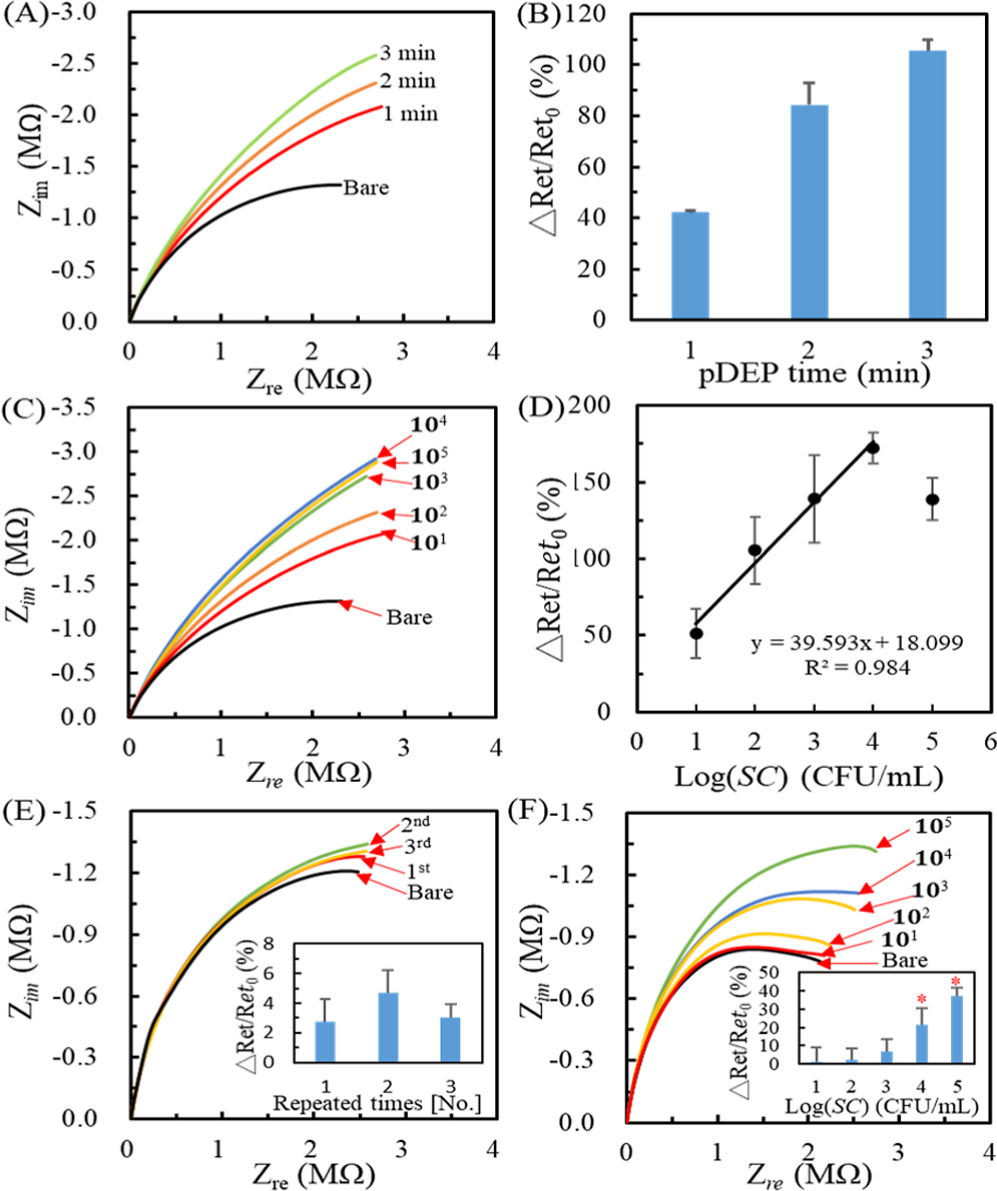
Effect of DEP collection time on the Nyquist plots (A) for the IMBs@*SC* (10^2^ cfu/mL) sample and the corresponding Δ*R*_et_/*R*_et0_ values (B). The Nyquist plots (C) and the calibration curve after conducting concentration-varied IMBs@*SC* solutions with the 3 min DEP collection at a flow rate of 25 μL/min. (D) The Δ*R*_et_/*R*_et0_ values correspond to (C). (E) The background test was conducted after only conducting the IMBs solution with the 3 min DEP. The inset shows the Δ*R*_et_/*R*_et0_ values. (F) The Nyquist plots after conducting concentration-varied IMBs@*SC* solutions without DEP collection under the control of a 25 μL/min flow rate. The inset shows the Δ*R*_et_/*R*_et0_ values. Each statistical data point is calculated from three individual WEs. The red asterisk indicates a significant difference (*p* < 0.05).

### Sensing Properties with 3 min DEP Collection

Figure 5C shows the Nyquist plots after conducting 100 μL concentration-varied IMBs@*SC* samples (10^1^–10^5^ cfu/mL) with the 3 min DEP collection. Figure 5D shows the calibration curve in the dynamic range of 10^1^–10^4^ cfu/mL with the regressive equation of Δ*R*_et_/*R*_et0_ (%) = 39.593 log­[*SC*] (cfu/mL) + 18.099 with a correlation coefficient (*R*) of 0.992, indicating good linearity. The calculated LOD was 2 cfu/mL. The lower LOD and the larger Δ*R*_et_/*R*_et0_ response to the same IMBs@*SC* concentration than those obtained from the 1 min DEP collection elucidate that the longer DEP time can effectively collect more IMBs@*SC* complexes to promote the EIS signal. However, Δ*R*_et_/*R*_et0_ declined at the IMBs@*SC* (10^5^ cfu/mL) concentration. It may be attributed to the excessive accumulation of the IMBs@*SC* complex in microholes, which causes large aggregates that easily escape from the microholes via fluidic shear force. In theory, each microhole, being a 10 μm diameter and 10 μm depth, is larger than that of a typical rod-shaped *SC*, 2–5 μm in length, and 0.5–1 μm in diameter. When the aggregate size of IMBs@SC complexes is larger than the microhole depth, the solution flow can remove the aggregate from the microhole, causing a smaller *R*_et_ signal.

Figure 5E shows the effect of the 3 min DEP on the collection of IMBs after conducting a 100 μL aliquot of 10^8^ IMBs/mL with a 25 μL/min flow rate. After regenerating the device and repeating the DEP collection, the EIS curves only slightly increased. The inset of Figure 5E shows the average Δ*R*_et_/*R*_et0_ values ranging from 2.8% to 4.7%, much smaller than the response of the IMBs@*SC* complexes. The phenomenon suggests that the nanometer-scaled IMBs suffer little pDEP force, which cannot capture them in the microholes because the pDEP force is proportional to the particle radius cubed.^45^ Therefore, the IMBs unbound to *SC* do not significantly affect the DEP and EIS results.

Figure 5F shows the gravity and fluidic effects on the sedimentation of IMBs@*SC* complexes without DEP collection in the microfluidic device. After the equivalent circuit simulation, the Δ*R*_et_/*R*_et0_ values are shown in the inset of Figure 5F. According to the student-*t* test calculation, Δ*R*_et_/*R*_et0_ values are significantly larger than the bare WEs only when the IMBs@*SC* concentration is above or equal to 10^4^ cfu/mL.

Moreover, the Δ*R*_et_/*R*_et0_ value of IMBs@*SC* (10^4^ cfu/mL) complexes with the 3 min DEP collection was 172.3 ± 10.2% (Figure 5D), which is 8.1 times larger than that (21.2 ± 9.4%) without the DEP collection. The results elucidate that the 3 min DEP collection can significantly improve *SC* detection sensitivity.

### Detection of *SC* in an Undiluted Milk Sample

Optimized parameters were applied to analyze actual undiluted milk samples spiked with *SC*. IMBs were incubated with the 100 or 200 μL aliquot of *SC*-spiked milk samples for 30 min. After magnetic separation, the IMBs@*SC* complexes were washed and resuspended in DI water. Figure 6A,C shows the Nyquist plots obtained from the 100 and 200 μL undiluted milk samples. Figure 6B,D shows their calibration curves. The results show that the sensitivity of regression equations is smaller than that obtained from the *SC*-spiked PBS (Figure 5D), which is attributed to the milk viscosity hindering the immunoreaction efficiency and magnetic collection. Moreover, the Δ*R*_et_/*R*_et0_ value of IMBs@*SC* (10^4^ cfu/mL) obtained from the 200 μL of milk is more significant than that obtained from the 100 μL of milk. The result implies that the increased milk volume can harvest more IMBs@*SC* to improve the EIS response. The calibration curve of Figure 6D exhibits a reasonable linear range of 10^1^ to 10^4^ cfu/mL with an *R* of 0.994. The regressive equation is Δ*R*_et_/*R*_et0_ (%) = 28.627 log­[*SC*] (cfu/mL) + 4.571 with a LOD of 2 cfu/mL (S/*N* > 3). Notably, the relative standard deviation of some data points of calibration curves in Figures 5 and 6 exceeds 15%, implying weak reproducibility. The phenomenon is attributed to our laboratory-level microfabrication techniques with a 2–5 μm resolution of SU-8 3010 photoresist, causing different WEs with different microhole areas. If industry-level microfabrications are adopted to reach 1 μm resolution, the reproducibility of microhole areas can be improved to obtain highly reproducible results.

**Figure 6:**
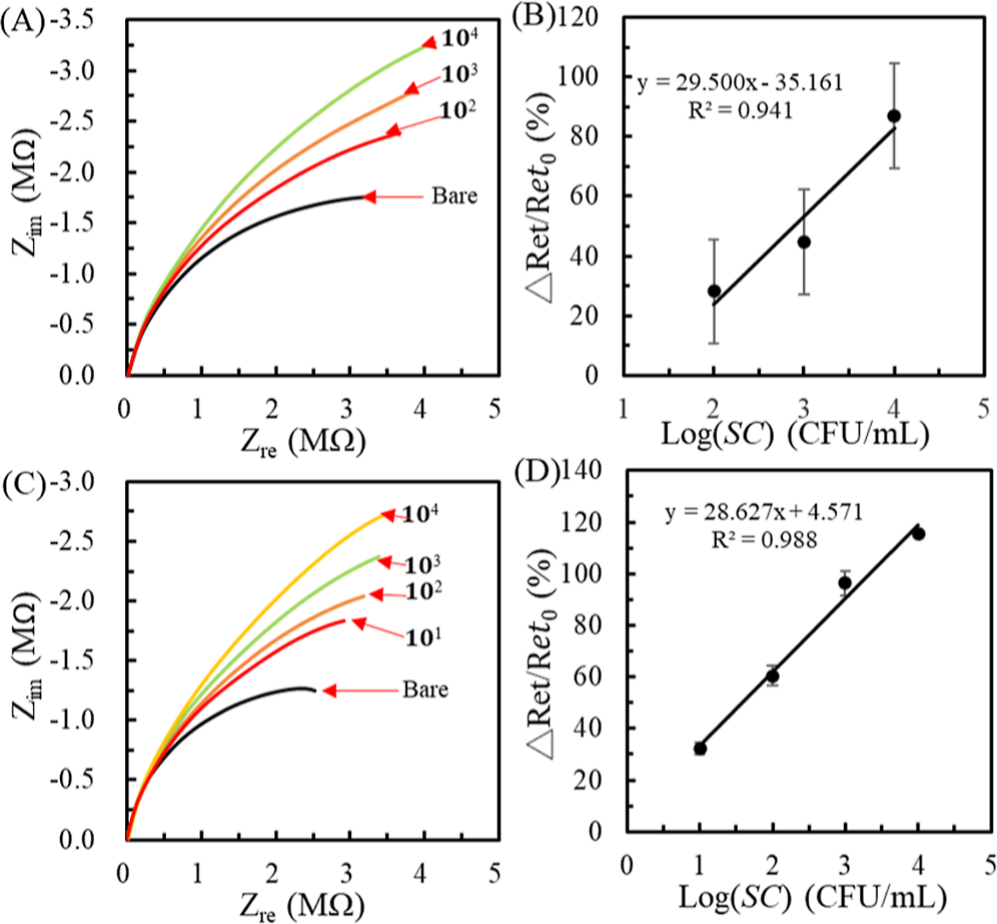
Nyquist plots of concentration-varied IMBs@*SC* complexes were collected from 100 (A) and 200 (B) μL of milk. (B and D) Calibration curves corresponding to (A and C). Each statistic data is calculated from three individual WEs.

The sensing characteristics of this study were compared with previous microfluidic bacteria-sensing devices integrating impedimetric or EIS detectors,^39,40,50–52^ as shown in Table S2 (Supporting Information). Notably, this study has the lowest LOD and a wide linear range. Moreover, our study is the first to integrate EIS measurement in a microfluidic device of DEP-assisted bacterial collection. In contrast to the antibody-modified interdigitated electrode, the electrodes have no complicated modification procedure. Our device integrating the microhole WEs only takes 3 min to complete the pDEP-based bacteria collection, which is shorter than the previous studies.^39,40,50–52^ The total analysis procedure from the IMBs-*SC* immunoreaction (30 min), magnetic separation (5 min), and DEP collection (3 min) to the EIS measurement (5 min) can be completed within 50 min. Furthermore, the ultrasensitive LOD of this sensing device permits direct *SC* detection from undiluted milk without bacterial preculture. Remarkably, utilizing IMBs can specifically separate target bacteria from actual liquid samples, such as milk, juice, and serum, saving the sample-to-result time of microfluidic devices.

## Conclusion

In this study, we constructed a microfluidic device with a top-bottom opposite electrode pair to perform the pDEP collection of IMBs@*SC* complexes and EIS measurement. The 50 kHz and 7.5 V_pp_ potential can generate a significant pDEP force to collect the micrometer-scale IMBs@*SC* complexes into the microholes of WE and act with little force on the unbound IMBs, which can effectively reduce the influence of unbound IMBs on the EIS signal. The Δ*R*_et_/*R*_et0_ values obtained from the EIS measurement can be used to quantify the IMBs@*SC* complex concentration. After the 3 min pDEP collection, the microfluidic sensing device had a wide linear range of 10^1^ to 10^4^ cfu/mL and an ultralow calculated 2 cfu/mL LOD. The results prove that the IMBs can successfully bind with *SC,* and the IMBs@*SC* complexes can be separated from the undiluted milk to fulfill actual sample detection. The total operation time from the IMBs immunoreaction to the EIS detection can be completed in 50 min. Moreover, the modification-free EIS detectors can be suitable for massive production by using microfabrication techniques and regenerated to extend their lifetime. The microfluidic device presents promising potential for urgent and sensitive bacterial detection.

## Supplementary Material



## Notes

Materials and methods; specificity of monoclonal 7F1A antibody; *Salmonella choleraesuis* preparation and fixation; preparation of IMBs@*SC* complex; activation of the microhole working electrode; results and discussion; and electric field simulation (PDF)

## Abbreviations

pDEP: positive dielectrophoresis
*SC*: *Salmonella enterica* serover Choleraesuis
IMBs: immunomagnetic beads
EIS: electrochemical impedance spectroscopy
cfu: colony-forming unit
LOD: limit of detection
WE: working electrode
RE and CE: reference and counter electrode
PBS: phosphate-buffered solution
FIC/FOC: K_3_[Fe (CN)_6_]/K_3_[Fe (CN)_6_]·3H_2_O
OD: optical density.
