# Supplementary material for: A Microfluidic Platform Integrating Dielectrophoretic Concentration and Impedimetric Sensing for Rapid Salmonella choleraesuis Detection
Source: ACS Sens. 2025 Sep 18;10(9):6512–20. doi: 10.1021/acssensors.5c00779 (PMC12481548; doi:10.1021/acssensors.5c00779)
Supplement: Supplementary file 1 [file se5c00779_si_001.pdf]

Supporting information

A Microfluidic Platform Integrating Dielectrophoretic Concentration and Impedimetric Sensing for Rapid *Salmonella Choleraesuis* Detection

Authors: Avinash V Police Patil [1, †], Yu-Sheng Chuang [1, †], Che-Wei Lin [1], Chiou-Ying Yang [2], Tomoyuki Yasukawa [3], Ching-Chou Wu [1, 4, *]

AUTHOR ADDRESS:

[1] Department of Bio-Industrial Mechatronics Engineering, National Chung Hsing University, Taichung City 402, Taiwan;

[2] Institute of Molecular Biology, National Chung Hsing University, Taichung City 402, Taiwan;

[3] Graduate School of Science, University of Hyogo, 3-2-1, Kouto, Kamigori, Ako, Hyogo, Japan;

[4] Innovation and Development Center of Sustainable Agriculture, National Chung Hsing University, Taichung City 402, Taiwan

[†] First authors have equal contribution

[*] Corresponding Author

E-mail Address: ccwu@dragon.nchu.edu.tw (C-C Wu) Tel.: +886-4-2285-1268 (Taiwan).

S2. Materials and Methods

S2.1. Specificity of Monoclonal 7F1A Antibody

By hybridoma technology, a monoclonal antibody (Ab, 7F1A) was generated in the laboratory of the coauthor, Dr. Chiou-Ying Yang. The anti-*Salmonella* Ab (7F1A) immunoreactivity testing was analyzed by enzyme-linked immunosorbent assay (ELISA), Western blotting, and immunofluorescence staining. The ELISA results in Figure S1 prove the 7F1A Ab with good immunoreactivity against *Salmonella enterica* subsp. *enterica* serovar Choleraesuis (*Salmonella Choleraesuis* (*SC*)). Figure S1(A) shows that the 7F1A Ab has better immunoreactivity for whole *SC* cells than the 5E6A anti-*Salmonella* Ab. Therefore, the 7F1A Ab was used in this study. Moreover, Fig. S1(B) shows the FITC-labeled detection Ab (DAb) immunofluorescence staining image to prove the 7F1A capture Ab (CAb) immune binding on *SC* cells simultaneously stained by the PI fluorescent indicator for double-strand DNA of dead *SC*. Figure S2 shows the Western blotting results using the 7F1A CAb against *Salmonella enterica* subsp. *enterica* serovar Enteritidis (*S. Enteritidis*), *Salmonella enterica* subsp. *enterica* serovar Typhimurium (*S. Typhimurium*), *Salmonella enterica* subsp. *enterica* serovar Typhi (*S. Typhi*), *Salmonella enterica* subsp. *enterica* serovar Minnesota (*S. Minnesota*), and *Escherichia coli* (*E. coli*). The results show that the 7F1A CAb exhibits a broad immunoactivity with different *Salmonella enterica* subsp. *enterica* serovars and a reasonable specificity without immunoreactivity with *E. coli*. The results of Figs. S1 and S2 demonstrate that the 7F1A CAb has broad immunoreactivity to whole bacterial cells (live and dead) and lysed bacteria.

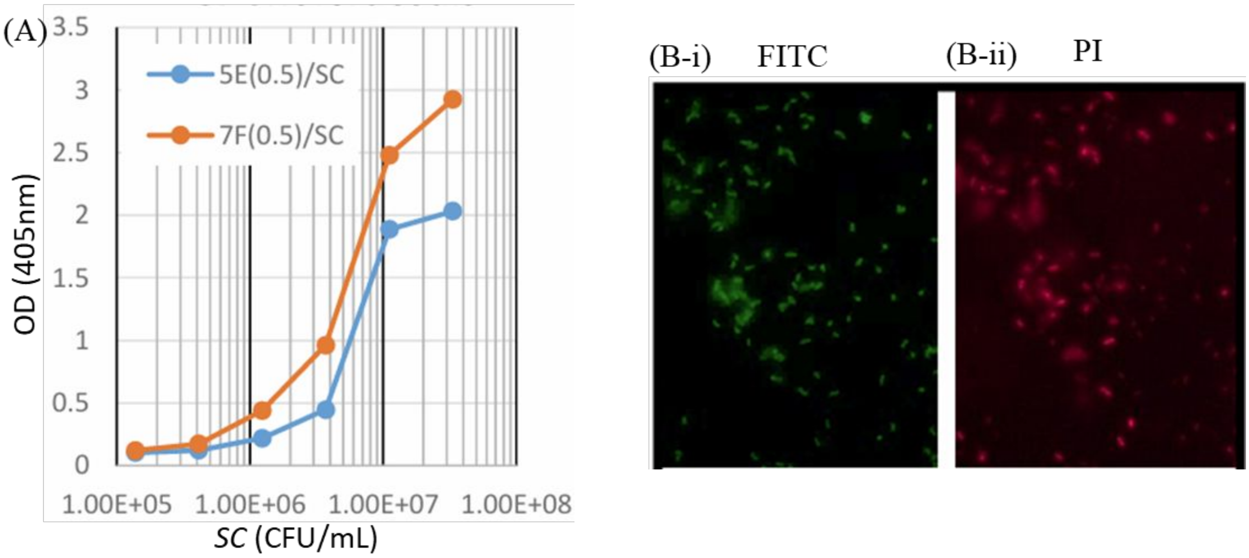

Figure S1 (A) Immunoreactivity test of two monoclonal anti-*Salmonella* CAb (5E6A and 7F1A) against *SC* using ELISA. Alkaline phosphatase (AP)-labeled DAb and nitrophenyl phosphate molecules were used to report the immunoreactivity between *SC* and the anti-*SC* CAbs via colorimetric measurement at 405 nm. (B) Fluorescent images of *SC* after staining FITC-labeled DAb (i) and PI (ii) with the 7F1A CAb-immunoreacted *SC*.

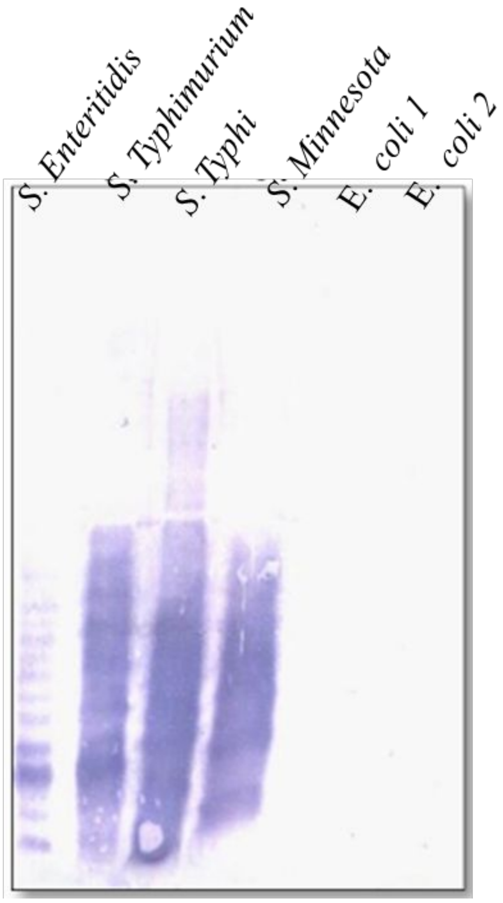

Figure S2 Western blotting results of different bacterial lysates immunoreacted by the 7F1A CAb and then alkaline phosphatase-labeled DAb. Colorimetric detection using 5-bromo-4-chloro-3-indolyl phosphate/nitro blue tetrazolium substrate for the AP-labeled DAb was employed to produce purple-blue bands on the membrane. *S. Enteritidis*: *Salmonella enterica* subsp. *enterica* serovar Enteritidis; *S. Typhimurium*: *Salmonella enterica* subsp. *enterica* serovar Typhimurium; *S. Typhi*: *Salmonella enterica* subsp. *enterica* serovar Typhi; *S. Minnesota*: *Salmonella enterica* subsp. *enterica* serovar Minnesota; *E. coli*: *Escherichia coli*.

Furthermore, scanning electron microscope (SEM) images were used to observe the magnetic collection efficiency via immunoreaction between the 7F1A CAb- and a monoclonal anti-*Acinetobacter baumannii* Ab(C12)-modified immunomagnetic beads (IMBs) and *SC*. The biotinylated 7F1A and C12 CAbs were bound on the streptavidin-modified MBs to form 7F1A-IMBs and C12-IMBs. Figures S3(A&B) respectively show *SC* and 7F1A-IMBs morphology. After magnetically collecting the immunoreaction products, the specific 7F1A-IMBs can significantly capture *SC* cells, as indicated with red arrows in Figure S3(C). In contrast, Figure S3(D) only show C12-IMBs without *SC* cells, implying that non-specific Ab-coated IMBs, like C12-IMBs, have no non-specific adsorption to the target *SC* cells after the magnetic collection procedures of IMBs. The results prove that the 7F1A-IMBs have good specificity for *SC,* and the IMBs collection doesn't cause non-specific affinity against non-target bacteria.

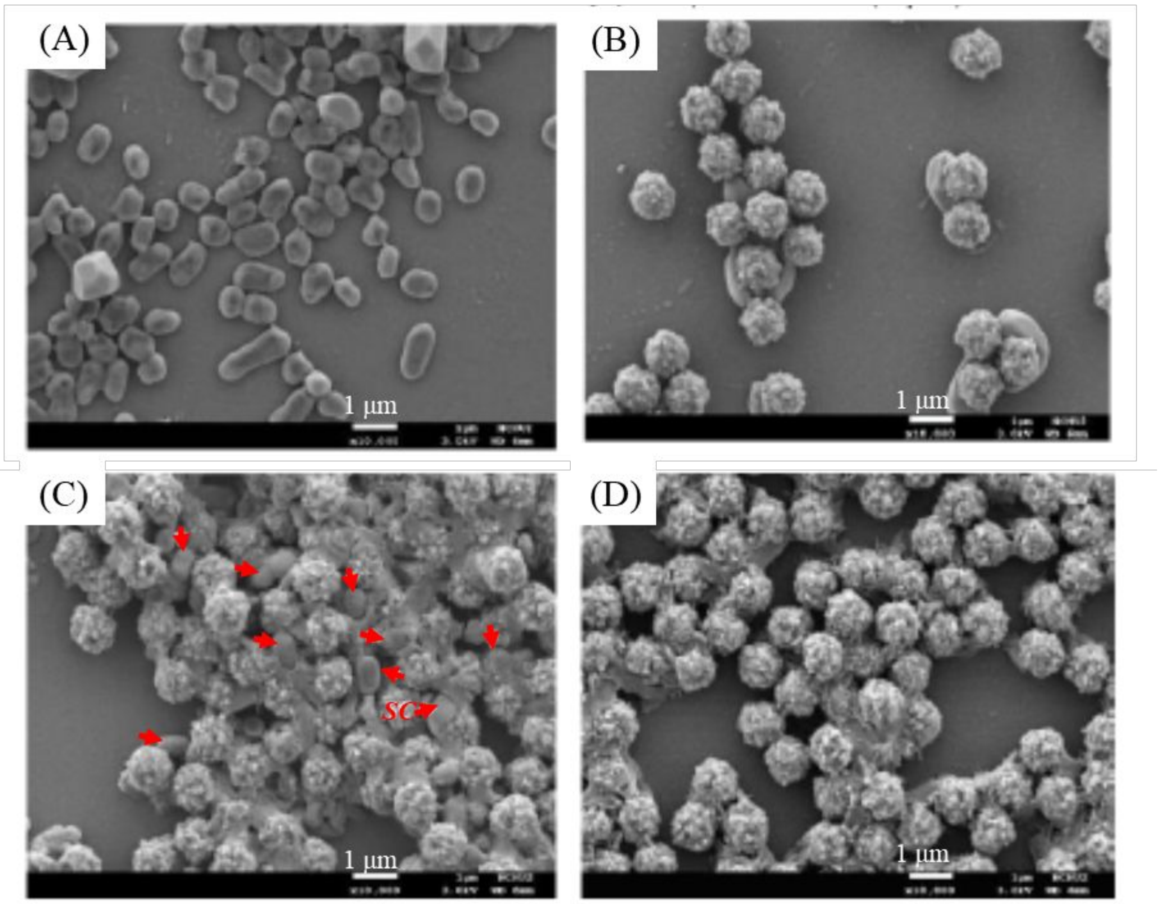

Figure S3 SEM images of (A) *SC*, (B) IMBs, (C) the 7F1A-IMBs, and (D) the C12-IMBs, respectively, immunoreacted with *SC* for 30 min and 5-min magnetic collection. The red arrows indicate *SC*.

S2.2. *Salmonella Choleraesuis* Preparation and Fixation

The *SC* strain was cultivated in Luria-Bertani (LB, DIFCO, Cat No. 244620) medium (LB broth). The cultivation conditions were at a constant temperature of 37°C with shaking at 150 rpm (FIRSTEL S300R). The bacterial solution turbidity was measured at 600 nm to reach a 1.0 OD value (around 1.8×10^9 CFU/mL). Then, the 1 mL aliquot of SC solution was centrifuged at 8000 rpm for 5 minutes, the supernatant was discarded, and the bacterial cells were resuspended in PBS. This process was repeated three times to wash off the LB broth. Finally, the bacterial cells were resuspended in 900 μL PBS fully. Then, a 100 μL aliquot of 37% formaldehyde (FA)(Sigma-Aldrich) was added to mix gently for 10 minutes at room temperature. Afterward, the bacterial cells were collected by 8000 rpm centrifugation for 5 minutes, the FA supernatant was removed, and PBS was added to suspend the bacteria. The procedure was repeated three times. Finally, the bacteria were resuspended in PBS to the required volume according to the experimental needs. We evaluated the immunoreactivity of monoclonal 7F1A Ab with *SC* OU7085 under non-fixed (-FA) and fixed (+FA) conditions using immunofluorescence staining, shown in Figure S4 (A&B). Bacterial cells were either untreated or treated with 3.7% formaldehyde for fixation before immunostaining with 7F1A Ab, followed by detection with FITC-conjugated anti-mouse IgG. The brightfield images confirmed the presence of bacterial cells (Figure S4 (A&B), left panels), while fluorescence images demonstrate specific antibody binding, with detectable signals observed under both conditions (Figure S4 (A&B), middle

panels). DAPI staining further confirmed the presence and distribution of *SC* cells (S4 (A&B), right panels). These results indicate that the 7F1A Ab effectively recognizes *SC* OU7085, regardless of fixation, making it a suitable candidate for immunodetection applications.

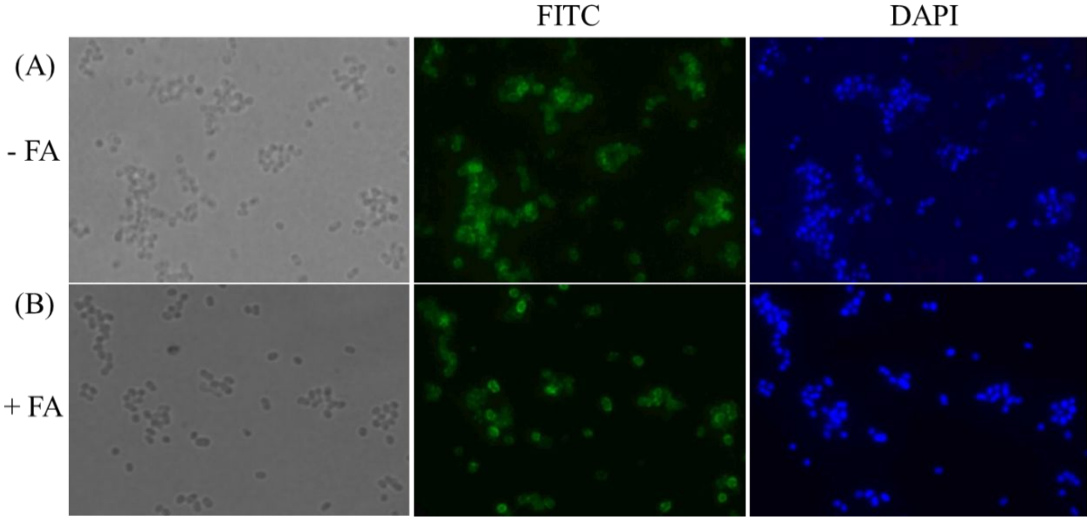

Figure S4 Immunofluorescence microscopy analysis of *SC* OU7085 stained with monoclonal 7F1A Ab under non-FA fixed (-FA, top row)(A) and FA fixed (+FA, bottom row) (B) conditions. Brightfield images (left) confirm bacterial morphology, FITC fluorescence images (middle) show mAb 7F1A binding detected using FITC-conjugated anti-mouse IgG, and DAPI fluorescence images (right) indicate bacterial nucleic acid staining. The results demonstrate specific antibody recognition of *SC* OU7085 under both fixation conditions.

S2.3. Preparation of IMBs@*SC*

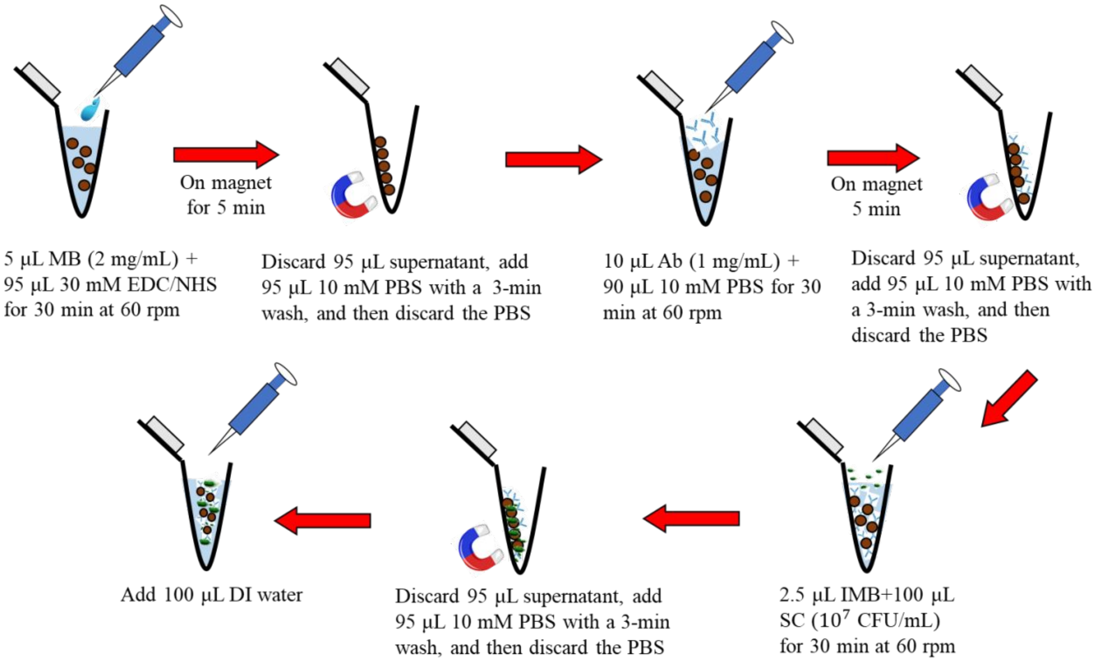

Figure S5 Schematic processes of IMB modification and their immunoreaction with *SC*.

S2.4. Activation of the micro-hole working electrode

The micro-hole WEs may produce gold oxide or suffer organic contamination to produce a passivation layer after being stored in the air. Before performing the electrochemical impedance spectrum (EIS) measurement of the positive dielectrophoresis (pDEP)-captured IMBs@*SC* complexes, the passivation layer of WEs should be removed to activate the electrode surface. Electrochemical cleaning is often used to oxidize the organic contaminant and reduce the metal oxide. The EIS was frequently used to estimate the electrochemical properties of the electrode/electrolyte interface. Figure S6 shows typical Nyquist plots of micro-hole WEs before and after the electrochemical cleaning of 50 kHz 7.5 V_{pp} potential for 5, 6, 7, and 8 min. The semicircle radius of Nyquist plots, implying the electron transfer resistance (R_{et}), drastically decreases after the five-minute potential application. The result indicates that the 7.5 V_{pp} potential can reduce the R_{et}. Furthermore, a time extension of 7.5 Vpp potentials up to 8 min did not cause the semicircle radius to change significantly, implying the 5-min 7.5 V_{pp} application has dramatically decreased the impedance of the WE surface.

The R_{et} value was obtained from the 1R//C equivalent circuit, consisting of one resistor (solution resistance (R_s)) in series with one parallel circuit comprising a resistor (R_{et}) and a constant phase element (CPE), which was mentioned in our previous articles to explain the kinetic behavior of the solution/electrode interface. Table S1 shows the corresponding R_{et} obtained from three micro-hole WEs fitted by the 1R//C equivalent circuit before and after the 7.5 V_{pp} application. The result indicates that the decrement ratios (=$R_{et\text{-}bare}$–$R_{et\text{-}5min}$/$R_{et\text{-}bare}$) of different WE's R_{et} significantly decrease in the range of 41.3%–55.9% after 5-min 7.5 V_{pp} application. Moreover, the later change in the R_{et} decrement ratios (=|$R_{et\text{-}5min}$–$R_{et\text{-}6,7,8min}$|/$R_{et\text{-}5min}$) from 5 to 8 min is only 1.1%–9.3%. The result suggests that the 5-min 7.5 V_{pp} pretreatment can effectively activate the micro-hole WEs to keep a stable R_{et} value. Moreover, the R_{et} decrement ratio is more adequate for evaluating the R_{et} change before and after using pDEP to capture IMBs@*SC* complexes in the micro-holes.

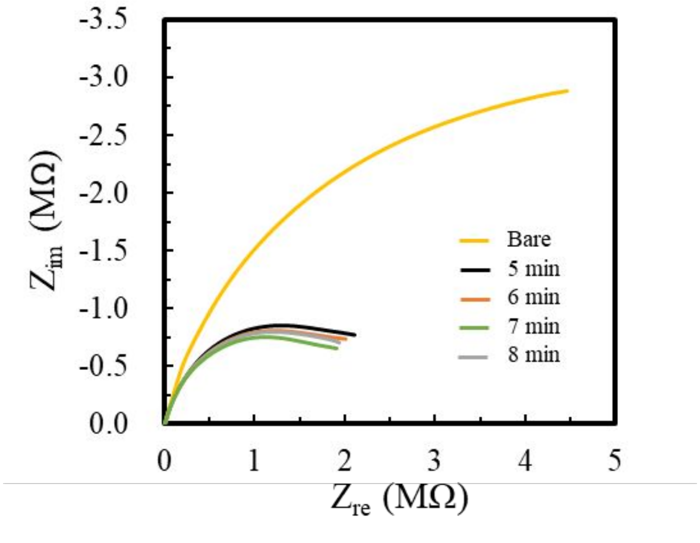

Figure S6 EIS measurement of micro-hole WE obtained in the 5 mM equimolar $[Fe(CN)_6]^{3-/4-}$-containing phosphate buffer solution before and after applying 50 kHz 7.5 V_{pp} potential for 5, 6, 7, and 8 min.

Table S1 The R_{et} values of different WEs fitted from the 1R//C equivalent circuit. Each measurement has three repetitions.

Electrode No.	R_{et} (MΩ) before and after 5-8 min electrochemical cleaning				
	Bare	5 min	6 min	7 min	8 min
WE1	4.75 ± 0.06	2.79 ± 0.07	3.05 ± 0.05	2.8 ± 0.04	2.76 ± 0.01
WE2	5.35 ± 1.17	2.36 ± 0.19	2.43 ± 0.08	2.31 ± 0.03	2.31 ± 0.05
WE3	8.79 ± 0.18	4.76 ± 0.09	5.09 ± 0.26	4.67 ± 0.12	5.16 ± 0.25

S3. Results and Discussions.

S3.1. Electric field simulation

The F_{DEP} is proportional to the del of the electric field square (∇E^2), and the pDEP drives conductivity-high particles to high electric field regions. We performed numerical simulations to visualize the electric field distribution with a channel height of 60 μm and three micro-holes (each 10 μm in diameter) patterned on the gold working electrode. The interval between adjacent micro-holes was fixed at 160 μm. Figure S7(A) presents the electric field distribution when +7.5 Vpp was applied to the Au electrode and 0 V to the ITO counter electrode. The results indicate that the highest electric field intensity occurs at the edge of each micro-hole, forming localized high-gradient zones suitable for pDEP trapping. Moreover, the 160 μm spacing between adjacent micro-holes helps create a steep field gradient between regions, enhancing dielectrophoretic selectivity. As shown in Figure S7(B), the electric field magnitude at the inner edge of a micro-hole reaches approximately 4.3×10^3 V/m, while the center of the interval between holes registers around 2.8×10^2 V/m. This contrast demonstrates a highly non-uniform electric field distribution, essential for inducing strong pDEP forces.

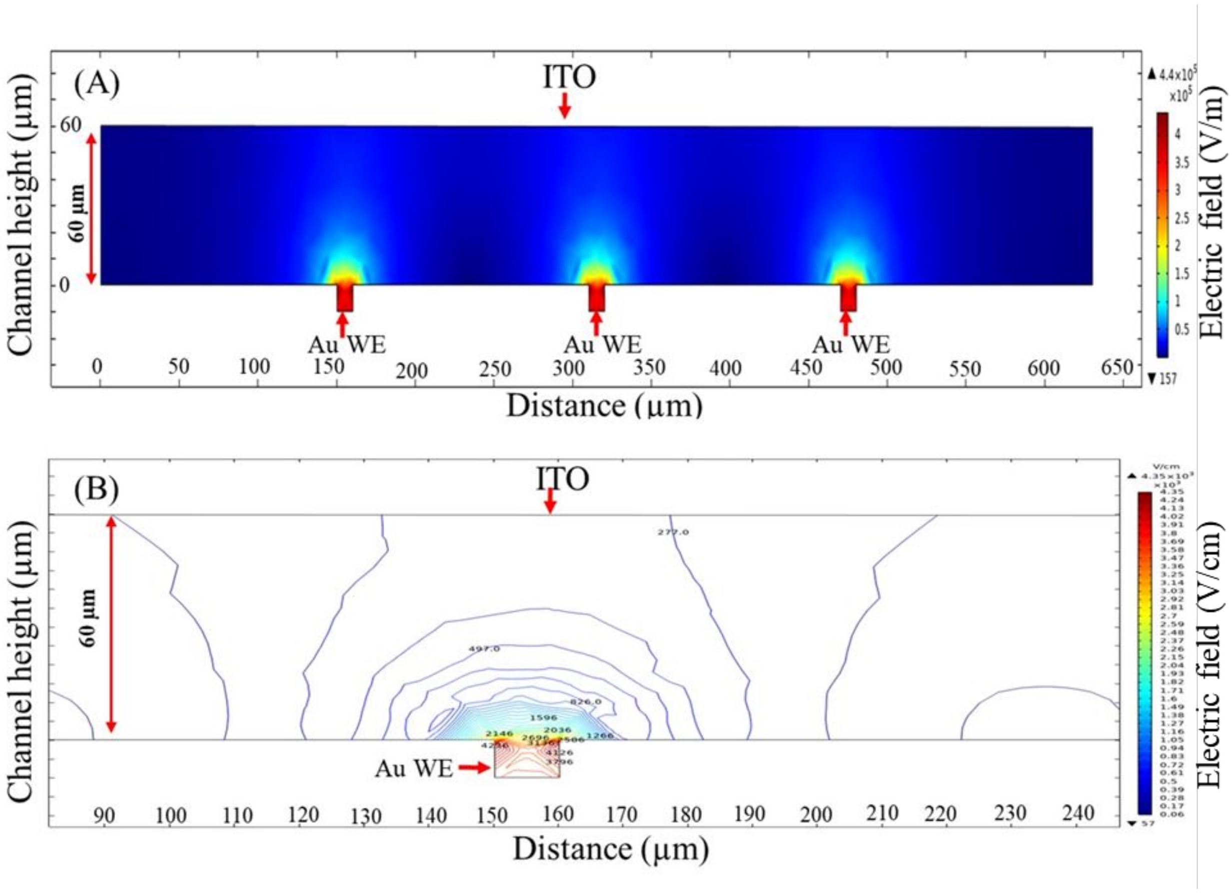

Figure S7 (A) The numerical simulation of the electric field in the microchannel. (B) Electric field contour values at a micro-hole working electrode (WE) neighborhood.

Table S2 Comparison with the sensing properties of present microfluidic devices integrating impedimetric or EIS detectors.

Electrode/Sensing strategy	Linear range (CFU/mL)	LOD (CFU/mL)	Time for electrode treatment/sample-to-result	Target collection techniques/Samples	Ref.
Label-free Si_3N_4-ITO IDE[1] /Impedance	5–5 × 10^3	36	None/40 min immunoreaction/2 min magnetic concentration and 2 min DEP collection	pDEP/IMBs@*Staphylo coccus* in 10% (w/v) homogenized cabbage solution	[39]
Label-free printed circuit board electrode/ Impedance	1.3 ×10^2–1.3 × 10^6 (medium)	53 (medium)	None/40 min immunoreaction/30 glucose catalysis	Magnetic force/IMBs@ *Salmonella*-glucose oxidase@polystyrene bead in medium and chicken meat	[40]
Antibody-modified IDE/Impedance	3–1000	3	165 min antibody immobilization/10 min immuno-reaction and 30 min washing	pDEP/*Salmonella, Legionella*, *Escherichia coli* in Tap water	[50]
Antibody-modified IDE/Impedance	7 – 125 (raw chicken) 8 – 120 (turkey)	7 (raw chicken) 8 (turkey)	75 min antibody immobilization/30 min immuno-reaction and 5 min washing	pDEP/*Salmonella* in turkey breast and raw chicken	[51]
Antibody-modified IDE/Impedance	10–120 (*Salmonella*) 13–1000 (*Escherichia coli*)	10 (*Salmonella*) 13 (*Escherichia coli*)	90 min antibody immobilization/30 min immuno-reaction and 30 min washing	pDEP/*Salmonella* and *Escherichia coli* in raw chicken	[52]
Label-free micro-hole WE/EIS	10–10^4	2	5 min activation/30 min immuno-reaction/3 min collection	pDEP/IMB@ *Salmonella* in undiluted milk	**This work**

[1]Si_3N_4-ITO IDE: silicon nitride-sputtered indium tin oxide interdigitated electrode;
